# Challenges in the diagnosis of the enigmatic primary adrenal leiomyosarcoma: two case reports and review of the literature

**DOI:** 10.1186/s12902-023-01530-z

**Published:** 2023-12-18

**Authors:** Sawako Suzuki, Naoya Takahashi, Masafumi Sugo, Kazuki Ishiwata, Akiko Ishida, Suzuka Watanabe, Katsushi Igarashi, Yutaro Ruike, Kumiko Naito, Masanori Fujimoto, Hisashi Koide, Yusuke Imamura, Shinichi Sakamoto, Tomohiko Ichikawa, Yoshihiro Kubota, Takeshi Wada, Yuto Yamazaki, Hironobu Sasano, Jun-ichiro Ikeda, Ichiro Tatsuno, Koutaro Yokote

**Affiliations:** 1 Department of Endocrinology, Hematology and Gerontology, Graduate School of Medicine, Chiba University, 1-8-1 Inohana, Chuo-ku, Chiba, 260-8670 Japan; 2 Department of Diabetes, Metabolism and Endocrinology, Chiba University Hospital, Chiba, Japan; 3 Department of Urology, Graduate School of Medicine, Chiba University, Chiba, Japan; 4 Department of Radiology, Chiba University Hospital, Chiba, Japan; 5 Department of Pathology, Tohoku University School of Medicine, Sendai, Japan; 6 Department of Diagnostic Pathology, Graduate School of Medicine, Chiba University, Chiba, Japan; 7 Chiba Prefectural University of Health Sciences, Chiba, Japan

**Keywords:** Primary adrenal leiomyosarcoma, CT-guided core needle biopsy, Vena cava thrombosis, Nonfunction, Case reports

## Abstract

**Background:**

Primary adrenal leiomyosarcoma is a rare and aggressive mesenchymal tumor derived from the smooth muscle wall of a central adrenal vein or its tributaries; therefore, tumors tend to invade the inferior vena cava and cause thrombosis. The great majority of tumors grow rapidly, which makes the disease difficult to diagnose in its early clinical stages and needs differentiation from adrenocortical carcinomas for the selection of chemotherapy including mitotane which causes adrenal insufficiency.

**Case presentation:**

We presented two patients with adrenal leiomyosarcoma who were referred to our hospital with abdominal pain and harboring large adrenal tumors and inferior vena cava thrombosis. The endocrine findings, including serum catecholamine levels, were unremarkable. These two patients were considered clinically inoperable, and CT-guided core needle biopsy was performed to obtain the definitive histopathological diagnosis and determine the modes of therapy. The masses were subsequently diagnosed as primary adrenal leiomyosarcoma based on the histological features and positive immunoreactivity for SMA (smooth muscle actin), desmin, and vimentin.

**Conclusions:**

Adrenal leiomyosarcoma derived from the smooth muscle wall of a central adrenal vein or its tributaries is rare but should be considered a differential diagnosis in the case of nonfunctioning adrenal tumors extending directly to the inferior vena cava. CT-guided biopsy is considered useful for histopathological diagnosis and clinical management of patients with inoperable advanced adrenal tumors without any hormone excess.

**Supplementary Information:**

The online version contains supplementary material available at 10.1186/s12902-023-01530-z.

## Background

Primary adrenal leiomyosarcoma is a rare mesenchymal tumor, representing 0.1% to 0.2% of all retroperitoneal soft tissue sarcomas of adults [1]. Primary adrenal leiomyosarcoma is well known to be derived from the smooth muscle wall of a central adrenal vein or its tributaries [2, 3], and the presence of tumor invasion extending from the central vein to the inferior vena cava resulting in thrombosis has been reported in those patients [4–12]. The great majority of adrenal leiomyosarcomas grow rapidly usually with nonspecific abdominal pain, and inoperable [13–17]. Therefore, differential diagnosis of the lesions is considered mandatory, especially needing differentiation from adrenocortical carcinomas for the selection of effective chemotherapy drugs including mitotane which causes adrenal insufficiency. We herein report two rare cases of inoperable primary adrenal leiomyosarcoma which were difficult to distinguish from adrenocortical carcinomas by clinical and imaging findings but only diagnosed by histopathological evaluation of CT-guided core needle biopsy.

## Case presentation

### Case 1

**Fig. 1:**
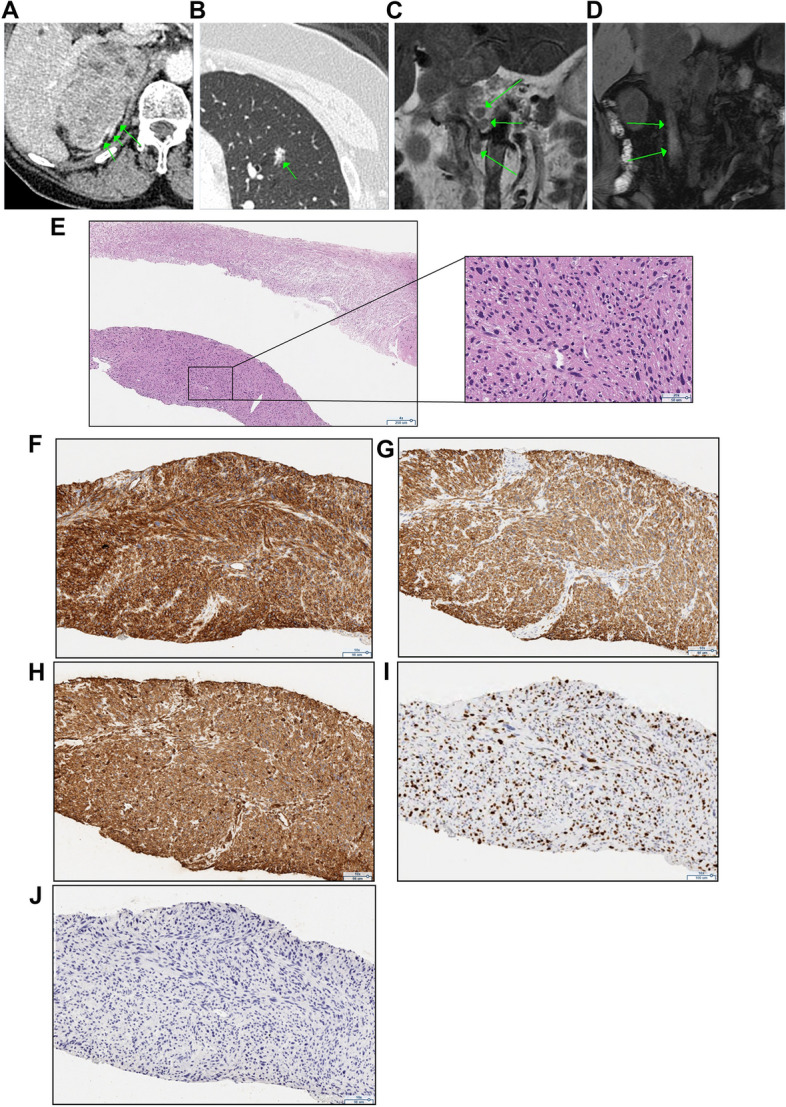
Radiological and histopathological characteristics in case 1. **A**-**B** An enhanced CT scan showing a 10 × 6 × 11 cm right-sided adrenal tumor with a normal adrenal gland (green arrow) on the dorsal side (**A**) and metastatic lesions in the lung (**B**). **C** T2-weighted MRI images showing lymphadenopathy in the hilum and para-aorta. **D** High signal in T1-weighted MRI images reveal venous thrombosis from the inferior vena cava to the right common iliac vein and the right internal iliac vein. **E** Hematoxylin eosin staining showing atypical spindle cells in adrenal biopsies (40 x). **F-H** Tumor cell staining positive for smooth muscle actin SMA (**F** 100 x), desmin (**G** 100 x) and vimentin (**H** 100 x). **I** Immunohistochemical staining of the cells with the Ki-67 marker (100 x). **J** EBV-encoded RNA in situ hybridization (EBER-ISH) staining for Epstein–Barr virus (EBV) was negative (100 x)

A 71-year-old woman with no past history was admitted to another hospital due to abdominal pain, and revealed to have a right retroperitoneal mass, thereby referred to our hospital. She has been treated with acetaminophen. At presentation, her blood pressure was slightly high (BP 144/78 mmHg) and body temperature was elevated (37.7 °C). Physical examination revealed a mild abdominal discomfort upon palpation. Routine laboratory investigation showed an average blood count (WBC 5800/µl) but high C-reactive protein levels (CRP: 20.16 mg/dL). Her HIV antibody test was negative. Only the tumor marker neuron-specific enolase (NSE: 44.6 ng/mL) was increased. All blood hormonal parameters were within normal limits: plasma cortisol 15.3 µg/dl (normal range 7.1–19.6), plasma adrenocorticotropic hormone (ACTH) 22.9 pg/ml (normal range 7.2–63.3), dehydroepiandrosterone sulfate (DHEAS) 29 µg/dl (normal range 7–177), aldosterone (RIA) 84.3 pg/ml (normal range 29.9–159), plasma renin activity 0.5 ng/ml/h (normal range 0.3–2.9), adrenaline 6 pg/ml (normal range < 100), noradrenaline 200 pg/ml (normal range 100–450) and dopamine 9 pg/ml (normal range < 20). 24-h urine collection for cortisol (50.3 µg/day: normal range 11.2–80.3), aldosterone (6.37 µg/day: normal range < 10), metanephrine (0.1 mg/day: normal range 0.04–0.19), normetanephrine (0.22 mg/day: normal range 0.09–0.33), and 17-ketosteroids (17-KS) were also within normal limits. Computed tomography (CT) demonstrated a poorly enhanced and heterogeneous mass measuring 10 × 6 × 11 cm in the right suprarenal area with a continuous normal adrenal gland on its dorsal side (Fig. 1A). In addition, there was an infiltration shadow on the bottom of the right lung (Fig. 1B), lymphadenopathy in the longitudinal, bilateral hilum, hilar, para-aorta, and inguinal regions (Fig. 1C), and obstruction of the inferior vena cava. Subsequent adrenal magnetic resonance imaging (MRI) showed an 11 cm heterogeneous mass with a lack of signal drop on out-of-phase imaging. A T1-weighted image showed a high signal from the inferior vena cava to the right common iliac vein and internal iliac vein, suggesting thrombosis (Fig. 1D). The metaodobenzylguanidine (MIBG) scintigram was negative. Because of distant metastasis to the lung and poor general condition, adrenalectomy could not be performed, and a CT-guided core needle biopsy using 16-gauge was subsequently performed. Histopathologically, spindle-shaped atypical cells harboring a high nuclear/cytoplasmic ratio were detected (Fig. 1E). The areas of necrosis and slight tumor heterogeneity and mitosis were seen in the pleomorphic areas. These atypical cells were immunohistochemically positive for smooth muscle actin (SMA) (Fig. 1F), desmin (Fig. 1G), and vimentin (Fig. 1H), consistent with the diagnosis of primary adrenal leiomyosarcoma. The Ki-67 proliferation index was 40% (Fig. 1I). The complete absence of immunoreactivity of SF-1 (Supplemental Fig. 1A), inhibin-alpha (Supplemental Fig. 1B), and calretinin (Supplemental Fig. 1C) ruled out adrenocortical carcinoma. In addition, the lack of synaptophysin, AE1/AE3 cytokeratin, S-100 and CD34 expression (Supplementary Fig. 1D-G) ruled out pheochromocytoma, metastatic carcinoma, malignant peripheral nerve sheath tumor, and angiosarcoma, respectively. The patient was also negative for Epstein–Barr virus (EBV), as demonstrated by EBV-encoded RNA (EBER) in situ hybridization (ISH) (Fig. 1J) and latent membrane protein 1 (LMP1) immunoreactivity (Supplemental Fig. 1H) despite the enlargement of multiple lymph nodes. The tumor cells were also immunohistochemically negative for p53 (Supplementary Fig. 1I). Doxorubicin chemotherapy was suggested, but the patient and her family chose not to undergo any additional chemotherapy or radiotherapy, and refractory pain control was performed by palliative care staff. The patient died 8 months after diagnosis.

### Case 2

**Fig. 2:**
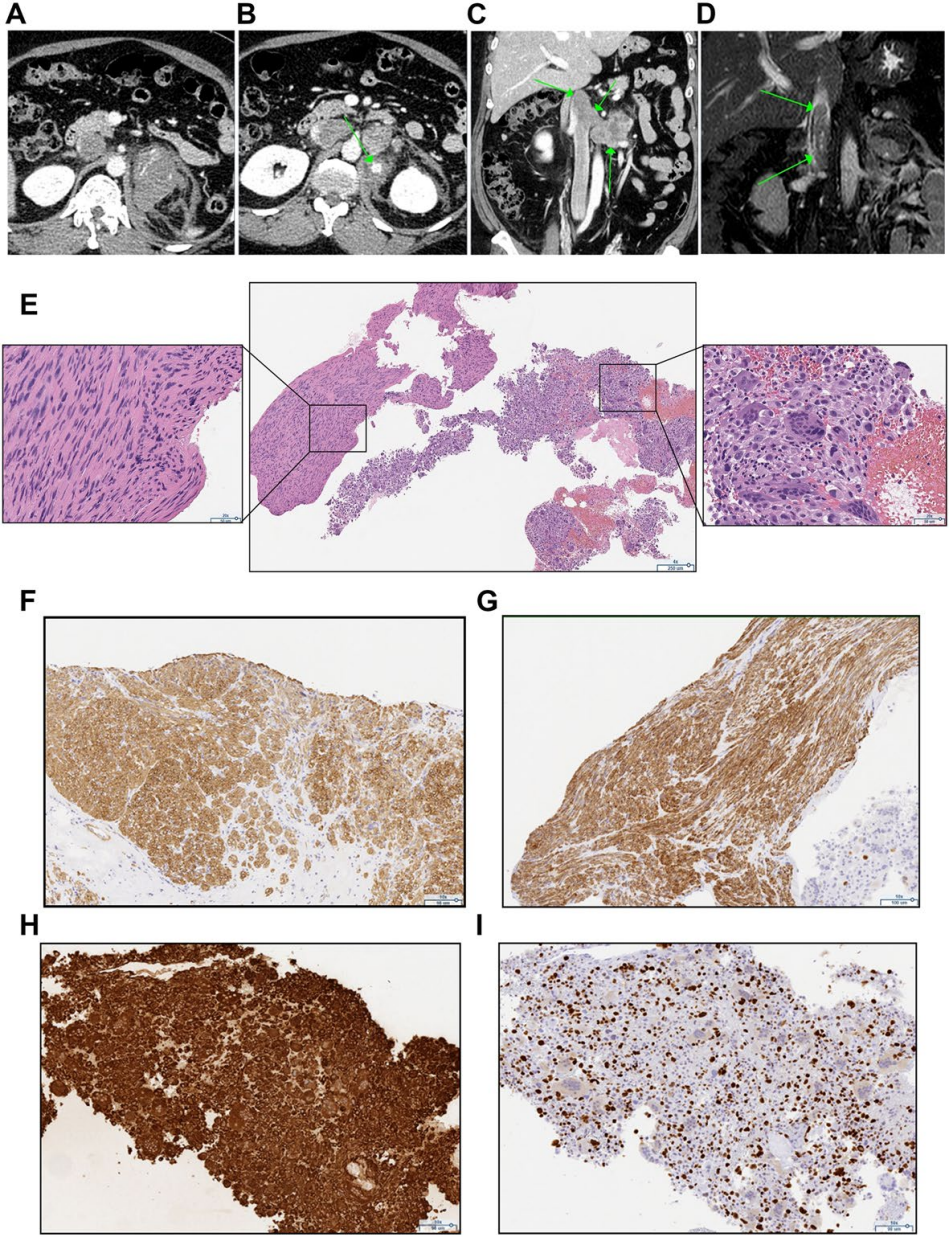
Radiological and histopathological characteristics in case 2. **A**-**C** An enhanced CT scan showing a 7 × 5 × 5 cm left-sided adrenal tumor with surrounding adipose tissue opacity, extravasation (**A**) and partial bleeding (**B**), as well as lymphadenopathy in the para-aortic region (**C**). **D** MRI revealed venous thrombosis extending from the left renal vein to the subhepatic vena cava. **E** Hematoxylin eosin staining showing atypical spindle cells and multinucleated cells in adrenal biopsies (40 x). **F**-**H** Tumor cell staining positive for smooth muscle actin SMA (**F** 100 x), desmin (**G** 100 x), and vimentin (**H** 100 x). **I** Immunohistochemical staining of the cells with the Ki-67 marker (100 x)

A 45-year-old woman with type 2 diabetes, hypertension and dyslipidemia was admitted to our hospital by ambulance with back pain, nausea, and cold sweats. At presentation, she had low blood pressure (BP 96/36 mmHg) and tachypnea (RR 48/min). She had an elevated body temperature (37.8 °C) and elevated white blood cells (10,200 cells/µL), CRP levels (16.84 mg/dL), and D-dimer (5.3 µg/ml). CT demonstrated a 7 × 5 × 5 cm solid mass with blurred boundaries and partial intratumoral bleeding in the left upper abdomen (Fig. 2A and B), accompanied by lymphadenopathy in the para-aortic region (Fig. 2C) and a tumor thrombus extending from the left adrenal vein to the renal and inferior vena cava, as demonstrated by MRI (Fig. 2D). All blood hormonal parameters were within normal limits: plasma cortisol 11.6 µg/dl (normal range 7.1–19.6), ACTH 10.0 pg/ml (normal range 7.2–63.3), DHEAS 125 µg/dl (normal range 19–231), plasma aldosterone (RIA) 74.3 pg/ml (normal range 29.9–159), plasma renin activity 1.0 ng/ml/hr (normal range 0.3–2.9), adrenaline 22 pg/ml (normal range < 100), noradrenaline 222 pg/ml (normal range 100–450) and dopamine 10 pg/ml (normal range < 20). 24-h urine collection for cortisol (97.7 µg/day: normal range 11.2–80.3), aldosterone (4.77 µg/day: normal range < 10), metanephrine (0.11 mg/day: normal range 0.04–0.19), normetanephrine (0.33 mg/day: normal range 0.09–0.33), as well as overnight 1-mg dexamethasone suppression test (1.7 µg/dl), were also within normal limits. The MIBG scintigram result was negative. The mass could not be surgically resected due to the tumor thrombus clinically detected in the left renal vein and inferior vena cava. A CT-guided core needle biopsy using a 16-gauge needle was subsequently performed to definitively diagnose the lesion. The tumor was composed of tumor heterogeneity of spindle-shaped atypical cells harboring a high nuclear/cytoplasmic ratio as well as nuclear pleomorphism with many multinucleated giant cells (Fig. 2E) immunohistochemically positive for SMA (Fig. 2F), desmin (Fig. 2G), and vimentin (Fig. 2H), consistent with the diagnosis of primary adrenal leiomyosarcoma. The absence of SF-1 (Supplemental Fig. 2A), chromogranin A/synaptophysin, AE1/AE3 cytokeratin, S-100, CD34, and HMB-45 (Supplemental Fig. 2B-G) ruled out pheochromocytoma, metastatic carcinoma, malignant peripheral nerve sheath tumor, angiosarcoma, and malignant melanoma, respectively. The Ki-67 index was 30% (Fig. 2I). p53 was detected in some tumor cells (Supplemental Fig. 2H). Chemotherapy with doxorubicin and ifosfamide was scheduled, but discontinued 1 month after diagnosis due to pulmonary embolus.

## Discussion and conclusions

**Fig. 3:**
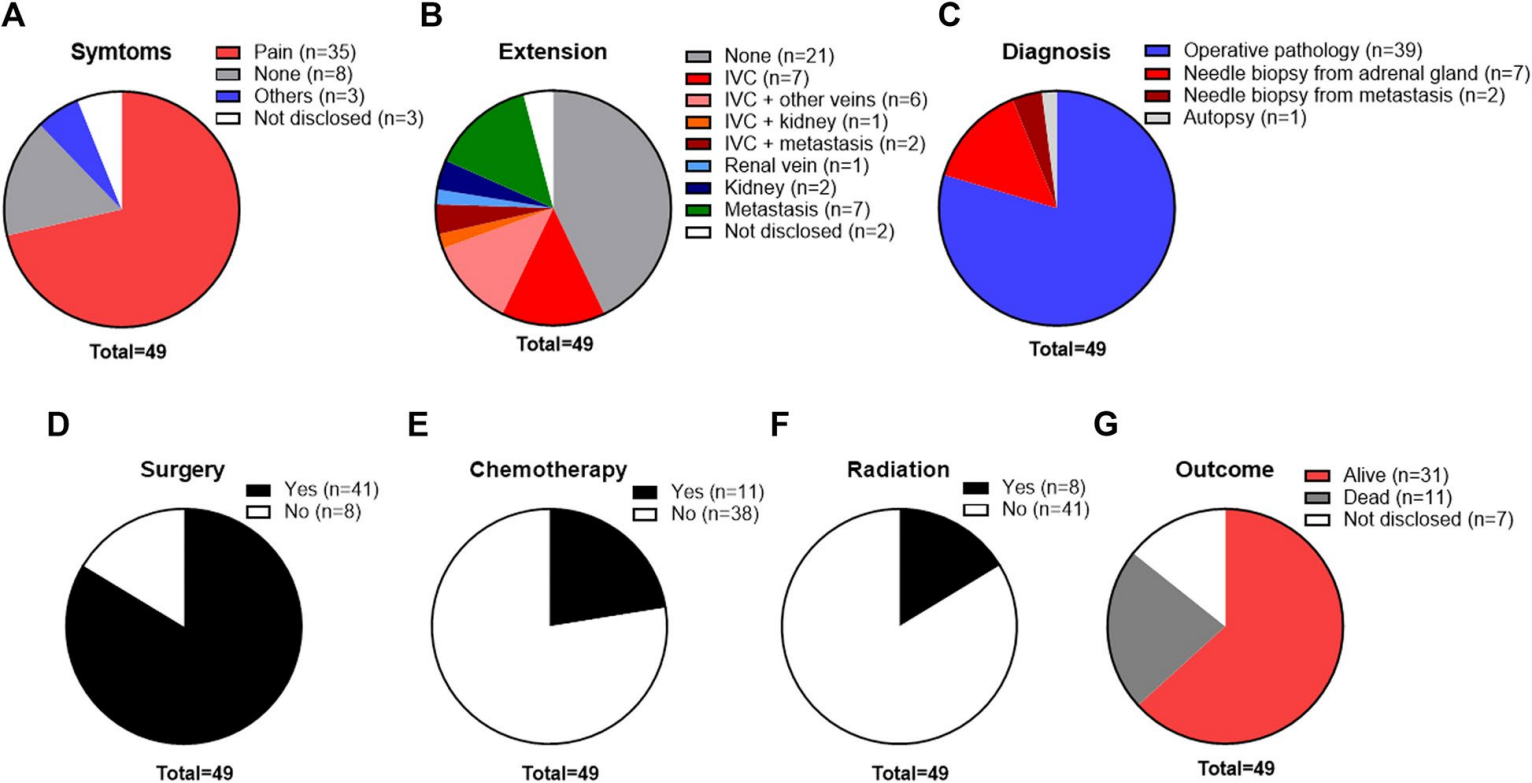
Analysis of the clinical features of the primary adrenal leiomyosarcoma, including previously reported cases and our two cases. A number of patients were analyzed in each clinical parameter. **A** Symptoms. **B** Metastatic lesions or local extensions. **C** Diagnosis procedures. **D**-**F** Patients treated with surgery (in **D**), chemotherapy (in **E**), and radiation (in **F**). **G** Patient outcomes

We presented two rare cases of primary adrenal leiomyosarcoma with an extremely poor prognosis. The primary adrenal leiomyosarcomas showed direct extension to the inferior vena cava, and could be distinguished from other retroperitoneal tumors including adrenocortical carcinoma, malignant pheochromocytoma and renal cell carcinoma [18] by only CT-guided core needle biopsy. The clinical and pathological features of the reported cases including our two cases are summarized in Table 1. The ages of the patients ranged from 14 to 79 years, with a mean of 54 years. The patients with primary adrenal leiomyosarcoma occur in 27 women and 22 men. There were 22 right-sided, 25 left-sided, and two bilateral tumors. The size at presentation has ranged from 0.8 to 27 cm (mean, 9.5 cm). The most common presenting symptom is pain (abdominal, flank, back, or groin) in 71.4% of patients (Fig. 3A). Primary adrenal leiomyosarcoma is well known to be derived from the smooth muscle wall of a central adrenal vein or its tributaries [2, 3]. The extension into the IVC was 32.6%: IVC alone 14.3%, IVC with right atrium or other veins (renal, iliac or hepatic) 12.2%, IVC with metastasis (lung, thoracic wall, femur or muscle) 4.1%, and IVC with a kidney 2% (Fig. 3B). The apparent invasion to the kidney and renal vein were 4.1% and 2%, respectively (Fig. 3B). The distal metastasis (liver, lung, bone, pancreas, and brain) was observed in 14.3% (Fig. 3B). The diagnosis of primary adrenal leiomyosarcoma was based on histopathological and immunohistochemical, showing neoplasm consisting of spindle cells that stain positively for SMA, desmin, vimentin, h-caldesmon or others (actin, keratin, cytokeratin, HHF, calpinin, NSE, CD163, MAK6, WT1 or S100). Most histopathological evaluation was performed after surgery in 79.6% of cases, but the needle biopsy from adrenal tumors was also performed in 14.3% and needle biopsy from metastasis (liver or lung) in 4.1% of cases (Fig. 3C). As for treatment, 16.3% of patients could not perform any surgery (Fig. 3D). The chemotherapy and/or radiation were performed in 22.4% and 16.3% of patients, respectively (Fig. 3E and F). During 11 days to 52 months follow-up duration, 63.3% of patients were alive and 22.4% were dead (Fig. 3G).

**Table 1:** Summary of the clinical and pathological features of primary adrenal leiomyosarcoma, including previously reported cases and our two cases

References		Patients’ characteristics			Tumors			Diagnosis	Pathology	Treatment	Outcome	Follow-up
(name)	(year)	Age (year)	Sex	symptoms	Side	Size (cm)	Extension	Procedure	Positive staining			Months
Our case		71	F	Pain (abdominal)	R	10	IVC	Needle biopsy (adrenal gland)	SMA, desmin and vimentin	None	Dead	8
Our case		45	F	Pain (back) and nausea	L	7	IVC and renal vein	Needle biopsy (adrenal gland)	SMA, desmin and vimentin	None	Dead	1
Lin H. [19]	2023	56	M	ND	R	7.4	IVC and renal vein	Needle biopsy (liver)	SMA and desmin	Adrenalectomy	Alive	6
Wang YH. [20]	2023	74	F	Pain (abdominal)	L	3.4	None	Operative pathology	SMA, desmin and h-caldesmon	Adrenalectomy	Alive	ND
Oshidari B. [21]	2022	32	F	Pain (abdominal)	R	10.6	IVC	Operative pathology	SMA, desmin and vimentin	Adrenalectomy	Alive	2
Waack A. [22]	2022	58	F	Pain (abdominal)	L	5.5	None	Operative pathology	SMA, desmin, vimentin, and h-caldesmon	Adrenalectomy	Alive	30
Wang Y. [23]	2020	29	F	None	R	3.4	None	Operative pathology	SMA, desmin, vimentin, and h-caldesmon	Adrenalectomy	Alive	12
Jabarkhel F. [24]	2020	58	F	Pain (abdominal)	L	6	Metastasis (liver)	Operative pathology	SMA, desmin, vimentin, and h-caldesmon	Chemotherapy	Alive	13
		66	M	None	R	7.5	IVC and metastasis (thoracic wall, femur and muscle)	Needle biopsy (adrenal gland)	SMA, desmin, h-caldesmon and CD163	Adrenalectomy and chemotherapy	Alive	23
		72	M	Pain (abdominal)	L	10	None	Operative pathology	SMA, desmin and h-caldesmon	Adrenalectomy and radiation	Alive	48
Sakellariou M. [25]	2020	62	M	None	L	10.3	Metastasis (bone, liver and pulmonary)	Operative pathology	SMA and desmin	Adrenalectomy, chemotherapy and radiation	Alive	31
Doppalapudi SK. [12]	2019	70	M	Abdominal varices	R	9	IVC and metastasis (lung)	Operative pathology	SMA, desmin, vimentin and h-caldesmon	Adrenalectomy + nephrectomy + thrombectomy	Dead	14
Nerli RB. [26]	2019	27	M	Pain (back)	L	9	None	Operative pathology	Desmin and h-caldesmon	Adrenalectomy	ND	ND
Mulani SR. [27]	2018	50	M	Pain (abdominal)	L	8.1	Metastasis (liver and lung)	Needle biopsy (adrenal gland)	Desmin, cytokeratin, MAK6, WT1 and S100	Chemotherapy and radiation	ND	ND
Onishi T. [28]	2016	34	M	Pain (abdominal)	R	5	IVC	Operative pathology	SMA	Adrenalectomy	Alive	10
Zhou Y. [2]	2015	49	F	Pain (back)	L	6	None	Operative pathology	SMA, desmin and vimentin	Adrenalectomy	Alive	6
Quildrian S. [29]	2015	44	F	Pain (abdominal)	R	12	None	Operative pathology	SMA, desmin, vimentin, h-caldesmon and HHF	Adrenalectomy	Alive	36
Nagaraj V. [1]	2015	61	M	Pain (flank)	L	17	None	Operative pathology	Desmin and vimentin	Adrenalectomy	ND	ND
Ozturk H. [9]	2014	70	F	Pain (flank)	R	8	IVC	Operative pathology	SMA and desmin	Adrenalectomy and chemotherapy	Alive	6
Lee S. [30]	2014	28	M	Pain (flank)	R	15	None	Operative pathology	SMA and desmin	Adrenalectomy	Alive	8
Gulpinar MT. [31]	2014	48	M	Lower urinary tract symptom	R	11	None	Operative pathology	SMA and vimentin	Adrenalectomy	Alive	8
Bhalla A. [17]	2014	45	M	Pain (abdominal)	R	11	Metastasis (liver)	Needle biopsy (adrenal gland)	Desmin and actin	Chemotherapy	Alive	9
Wei J. [32]	2014	57	F	None	L	8	None	Operative pathology	SMA, desmin, vimentin and actin	Adrenalectomy	Alive	29
Alam MM. [33]	2014	35	F	Pain (flank)	L	8.5	None	Operative pathology	ND	Adrenalectomy	ND	ND
Deshmukh SD. [34]	2013	60	F	Pain (flank)	L	5	None	Operative pathology	SMA, desmin and vimentin	Adrenalectomy	Alive	21
Liu SV. [35]	2012	79	F	Pain (abdominal)	L	6.3	ND	Operative pathology	ND	Adrenalectomy	Alive	12
Shao IH. [8]	2012	66	M	Abdominal fullness and nausea	L	10	Renal vein	Operative pathology	SMA and desmin	Adrenalectomy	Alive	18
Kanthan R. [36]	2012	28	F	Pain (abdominal)	L	16	Kidney	Operative pathology	SMA and vimentin	Adrenalectomy + nephrectomy	ND	ND
Karaosmanoglu A. [16]	2010	63	M	Pain (abdominal)	R	ND	IVC	Needle biopsy (adrenal gland)	Desmin, vimentin, actin and keratin	Chemotherapy	Dead	3
Hamada S. [37]	2009	62	F	Pain (flank)	Bil	8	None	Operative pathology	SMA	Adrenalectomy, chemotherapy and radiation	Dead	16
Van Laarhoreu HW. [15]	2009	78	M	Pain (abdominal)	L	ND	Metastasis (lung, pancreas, bone and brain)	Needle biopsy (adrenal gland)	SMA, vimentin and actin	Radiation	Dead	11 days
Goto J	2008	73	F	Pain (flank)	R	8	IVC and kidney	Operative pathology	SMA and NSE	Adrenalectomy + nephrectomy	Alive	10
Mencoboni M. [38]	2008	75	F	None	R	8	None	Operative pathology	SMA, desmin and actin	Adrenalectomy	Alive	12
Mohanty SK. [39]	2007	47	F	Pain (abdominal)	L	10	None	Operative pathology	Desmin, calpinin and actin	Adrenalectomy + nephrectomy and radiation	Alive	9
Wang TS. [7]	2007	64	F	None	R	14	IVC and right atrium	Operative pathology	SMA and desmin	Adrenalectomy + thrombectomy	Alive	10
Lee CW. [40]	2006	49	M	Pain (flank)	L	3	None	Operative pathology	Desmin	Adrenalectomy	Alive	10
Wong C. [6]	2005	57	M	Pain (groin)	L	ND	IVC and both iliac veins	Operative pathology	ND	Adrenalectomy + nephrectomy + thrombectomy	Dead	6
Candanedo-Gonzalez FA. [41]	2005	59	F	Pain (flank) and weight loss	L	16	Metastasis (liver)	Operative pathology	Desmin, vimentin and actin	Adrenalectomy, chemotherapy and radiation	Alive	36
Kato T. [5]	2004	59	M	ND	L	10	IVC	Operative pathology	SMA, desmin and vimentin	Adrenalectomy + nephrectomy + thrombectomy	Dead	6
Linos D. [42]	2004	14	F	None	Bil	3.5	None	Operative pathology	SMA, vimentin, actin and HHF	Adrenalectomy	ND	ND
Thamboo TP. [43]	2003	68	F	Pain (loin) and fever	R	13	None	Operative pathology	SMA, desmin, vimentin and actin	Adrenalectomy + nephrectomy + hepatic lobectomy + cholecystectomy and chemotherapy	Alive	12
Lujan MG. [44]	2003	63	M	None	R	25	Metastasis (lung, liver and kidney)	Needle biopsy (lung)	ND	Adrenalectomy + nephrectomy	Dead	shortly
Matsui Y. [4]	2002	61	F	Pain (flank) and fever	R	ND	IVC and right atrium	Operative pathology	SMA	Adrenalectomy + nephrectomy + thrombectomy	Dead	1
Etten B. [14]	2001	73	F	Pain (abdominal)	R	27	IVC	Operative pathology	SMA	Exploratory laparotomy	Dead	3 weeks
Boman F. [13]	1997	29	M	Autopsy	L	0.8	None	Autopsy	SMA and HHF	None	ND	ND
Zetler PJ. [45]	1995	30	M	Pain (abdominal)	L	11	ND	Operative pathology	SMA	Adrenalectomy	Alive	20
Hayashi J. [46]	1995	55	F	Pain (abdominal) and fever	R	ND	IVC, hepatic vein and right atrium	Operative pathology	ND	Adrenalectomy + nephrectomy	Alive	52
Lack EE. [3]	1991	49	M	Pain (flank)	R	11	None	Operative pathology	SMA, vimentin and actin	Adrenalectomy + nephrectomy, chemotherapy and radiation	Alive	9
Choi SH. [47]	1981	50	F	Pain (flank)	L	16	Kidney	Operative pathology	ND	Adrenalectomy + nephrectomy	Alive	12

*F* Female, *M* Male, *R* Right, *L* Left, *Bil* Bilateral, *ND* Not disclosed, *IVC* Inferior vena cava, *SMA* Smooth muscle actin

The clinical utility of adrenal biopsy including CT-guided core needle biopsy for other than adrenal lymphoma has been in dispute for a number of years. For instance, The European Society of Endocrinology Clinical Practice guidelines recommend against the use of an adrenal biopsy in the diagnostic work-up of patients suspected to harbor adrenocortical carcinoma unless there is sufficient evidence of metastatic disease that precludes surgery, and histopathologic proof is definitively required to determine the clinical management of the patients [48] because of its relatively high nondiagnostic rate (8.7%) and the overall rate of complications such as pneumothorax, pain, and adrenal hemorrhage (2.5%) [49, 50]. However, some patients with newly diagnosed single, large adrenal masses without other primary cancers have obtained enormous clinical benefits after undergoing adrenal biopsy [51]. Notably, approximately 30–50% of patients with adrenocortical carcinoma have no endocrinological abnormalities [51], and in those cases, the differential diagnosis of the lesions is mandatory to define their clinical management. For instance, mitotane therapy in conjunction with chemotherapy can be administered only for adrenocortical carcinoma patients and not for those with other adrenal lesions [52, 53], although its side effects are clinically not negligible [54]. In our present study, the two patients were deemed clinically inoperable, and appropriate diagnosis and subsequent therapeutic decisions could be achieved only by CT-guided core needle biopsy results. The tumor cells were composed of intersecting and sharply margined fascicles of atypical spindled immunohistochemically positive for SMA, desmin and vimentin, yielding the final diagnosis of primary adrenal leiomyosarcoma. Some cases of primary adrenal leiomyosarcoma were reported to be associated with high serum NSE levels [55–57]; however, serum NSE levels were measured in only one case in this study and were not high, and further investigations are required for clarification. In addition, of particular interest, HIV or EBV infection has been reported to be involved in the development of primary adrenal leiomyosarcoma because some primary adrenal leiomyosarcoma occurred in an immunosuppressive situation [13, 45, 58]. However, case 1 in our present study harboring bilaterally symmetric lymphadenopathy was negative for HIV and EBV; thus, the involvement of these infections requires further investigation.

In conclusion, adrenal leiomyosarcomas are malignant tumors derived from the smooth muscle cells in the wall of the central adrenal vein or its tributaries, and should be considered in cases of nonfunctioning adrenal tumors associated with direct extension from adrenals to the inferior vena cava. Primary adrenal leiomyosarcoma proliferates rapidly and is generally difficult to diagnose early; therefore, CT-guided core needle biopsy is considered a clinically useful approach for patient management.

### Supplementary Information

**Additional file 1: Supplemental Figure 1.** The immunohistochemical analysis of samples from case 1. **A-I** Negative staining of SF-1 (**A** 100 x), inhibin-alpha (**B** 100 x), calretinin (**C** 100 x), synaptophysin (**D** 100 x), CK AE1/AE3 (**E **100 x), S-100 (**F** 100 x), CD34 (**G** 100 x), latent membrane protein 1 (LMP1) (**H** 100 x), and p53 (I 100 x). **Supplemental Figure 2.** The immunohistochemical analysis of samples from case 2. **A-H** The negative staining of SF-1 (**A **100 x), chromogranin A (**B** 100 x), synaptophysin (**C** 100 x), CK AE1/AE3 (**D** 100 x), S-100 (**E** 100 x), CD34 (**F** 100 x), and HMB-45 (**G** 100 x). p53 staining was positive in some tumour cells (**H** 100 x).

## Data Availability

Data sharing is not applicable to this article as no datasets were generated or analyzed during the study.

## Abbreviations

SMA: Smooth muscle actin
CRP: C-reactive protein levels
NSE: Neuron-specific enolase
MIBG: Metaodobenzylguanidine
EBV: Epstein–Barr virus
EBER: EBV-encoded RNA
ISH: In situ hybridization
LMP1: Mlatent membrane protein 1
