# Supplementary material for: Challenges in the diagnosis of the enigmatic primary adrenal leiomyosarcoma: two case reports and review of the literature
Source: BMC Endocr Disord. 2023 Dec 18;23:276. doi: 10.1186/s12902-023-01530-z (PMC10726553; doi:10.1186/s12902-023-01530-z)
Supplement: Supplementary file 1 — Additional file 1: Supplemental Figure 1. The immunohistochemical analysis of samples from case 1. A-I Negative staining of SF-1 (A 100 x), inhibin-alpha (B 100 x), calretinin (C 100 x), synaptophysin (D 100 x), CK AE1/AE3 (E 100 x), S-100 (F 100 x), CD34 (G 100 x), latent membrane protein 1 (LMP1) (H 100 x), and p53 (I 100 x). Supplemental Figure 2. The immunohistochemical analysis of samples from case 2. A-H The negative staining of SF-1 (A 100 x), chromogranin A (B 100 x), synaptophysin (C 100 x), CK AE1/AE3 (D 100 x), S-100 (E 100 x), CD34 (F 100 x), and HMB-45 (G 100 x). p53 staining was positive in some tumour cells (H 100 x). [file 12902_2023_1530_MOESM1_ESM.docx]

**Supplementary Information for**

**Challenges in the diagnosis of the enigmatic primary adrenal leiomyosarcoma: two case reports and review of the literature.**

Sawako Suzuki^1,2*^, Naoya Takahashi^2^, Masafumi Sugo^1,2^, Kazuki Ishiwata^1,2^, Akiko Ishida^1,2^, Suzuka Watanabe^1,2^, Katsushi Igarashi^1,2^, Yutaro Ruike^1,2^, Kumiko Naito^1,2^, Masanori Fujimoto^1,2^, Hisashi Koide^1,2^, Yusuke Imamura^3^, Shinichi Sakamoto^3^, Tomohiko Ichikawa^3^, Yoshihiro Kubota^4^, Takeshi Wada^4^, Yuto Yamazaki^5^, Hironobu Sasano^5^, Jun-ichiro Ikeda^6^, Ichiro Tatsuno^7^, Koutaro Yokote^1,2^

^1^Department of Endocrinology, Hematology and Gerontology, Graduate School of Medicine, Chiba

University, Chiba, Japan.

^2^Department of Diabetes, Metabolism and Endocrinology, Chiba University Hospital, Chiba, Japan.

^3^Department of Urology, Graduate School of Medicine, Chiba University, Chiba, Japan.

^4^Department of Radiology, Chiba University Hospital, Chiba, Japan.

^5^Department of Pathology, Tohoku University School of Medicine,

^6^Department of Diagnostic Pathology, Graduate School of Medicine, Chiba University, Chiba, Japan.

^7^Chiba Prefectural University of Health Sciences.

**Short title**: Adrenal leiomyosarcoma with vena cava thrombosis.

**Keywords**: Primary adrenal leiomyosarcoma, CT-guided core needle biopsy, vena cava thrombosis, nonfunction.

**^*^Correspondance:** Sawako Suzuki, MD, PhD, Department of Endocrinology, Hematology and Gerontology, Chiba University Graduate School of Medicine, 1-8-1 Inohana, Chuo-ku, Chiba 260-8670, Japan. Phone: 81-43-226-2092; Fax: 81-43-226-2095. E-mail; sawakosuzuki@chiba-u.jp

This word file includes:　Supplemental Figure 1 and 2.


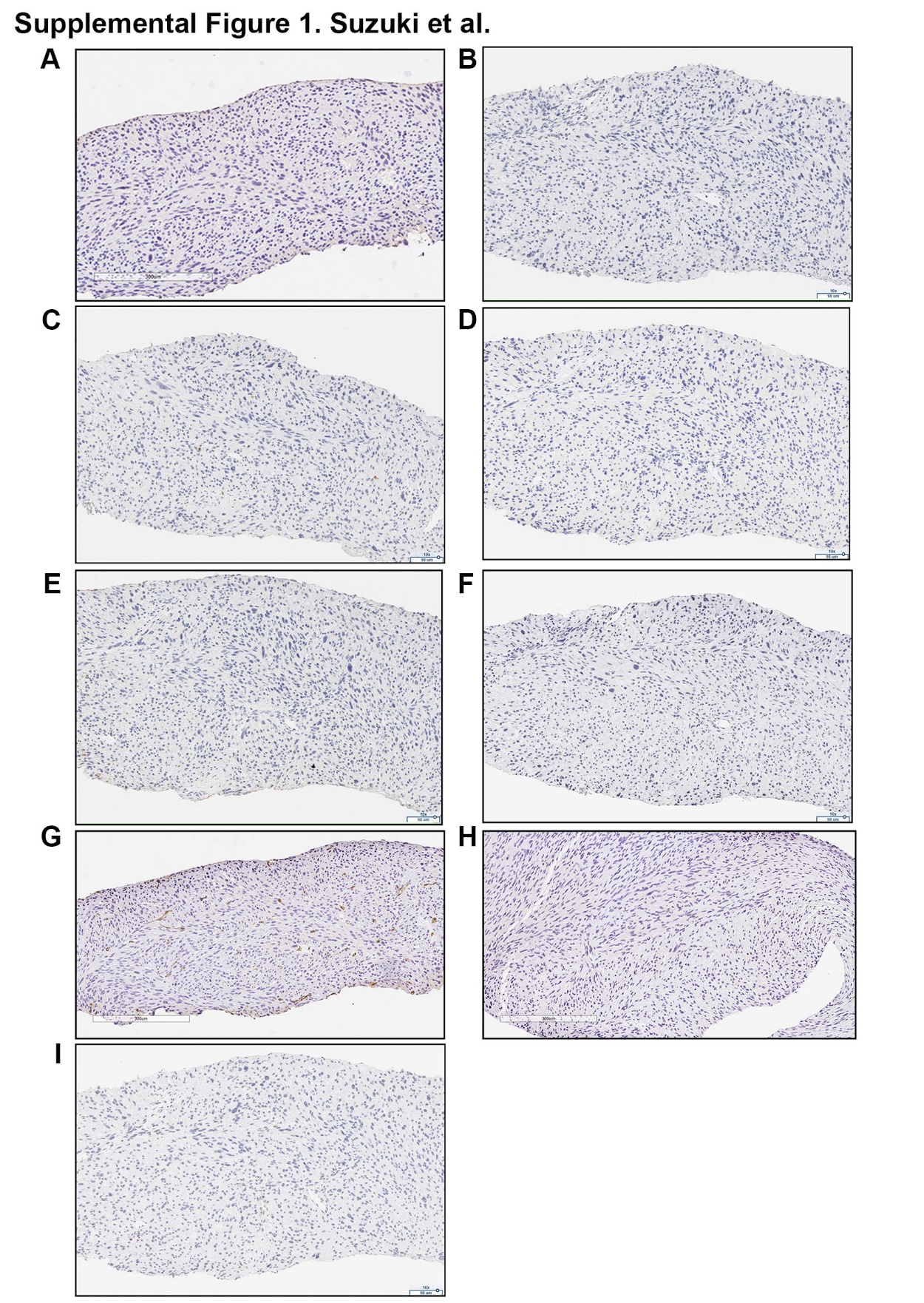


**Supplemental Figure 1.** The immunohistochemical analysis of samples from case 1. (A-I; 100 x) Negative staining of SF-1 (A), inhibin-alpha (B), calretinin (C), synaptophysin (D), CK AE1/AE3 (E), S-100 (F), CD34 (G), latent membrane protein 1 (LMP1) (H), and p53 (I).


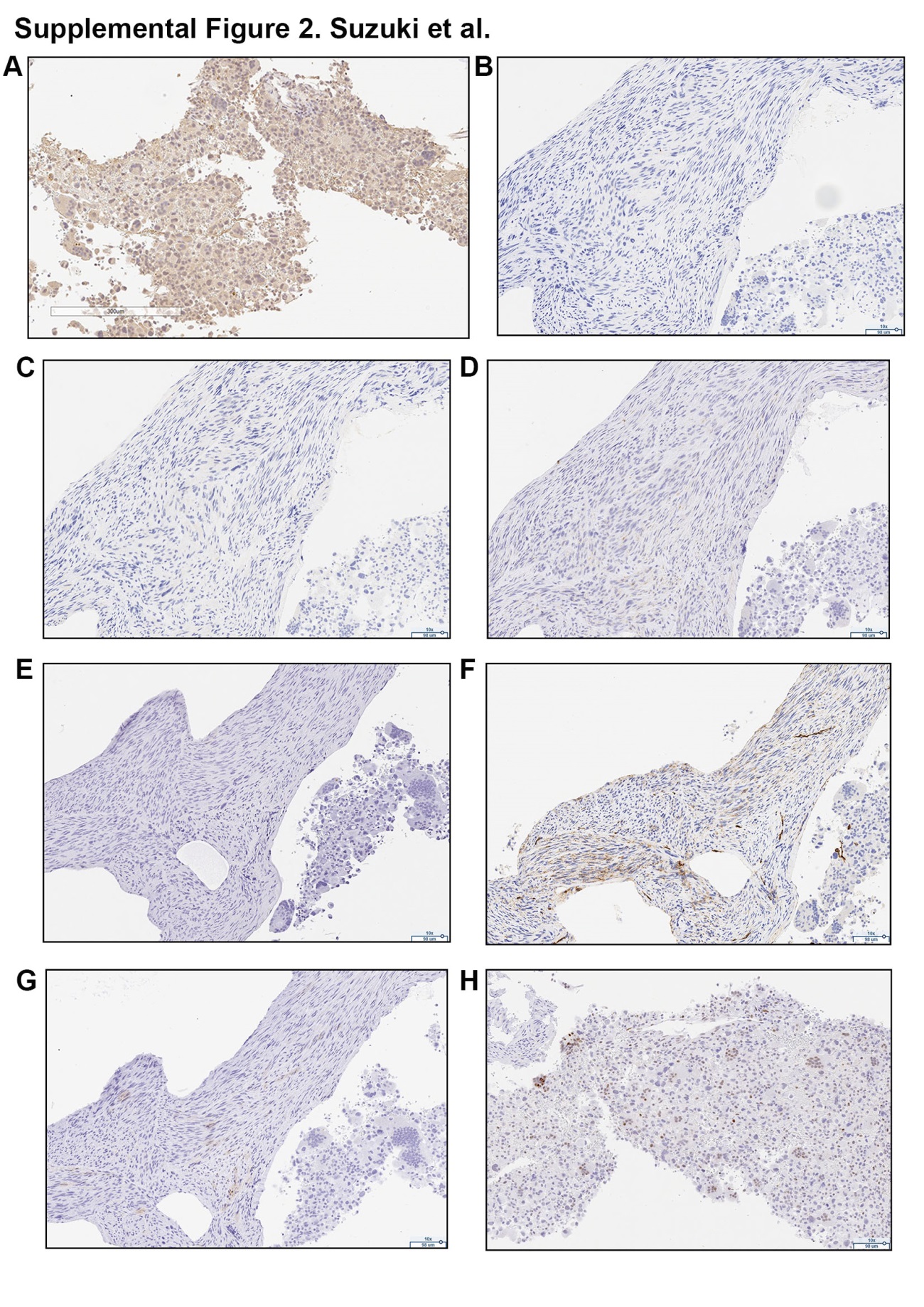


**Supplemental Figure 2.** The immunohistochemical analysis of samples from case 2. (A-H; 100 x) The negative staining of SF-1 (A), chromogranin A (B), synaptophysin (C), CK AE1/AE3 (D), S-100 (E), CD34 (F), and HMB-45 (G). p53 staining was positive in some tumour cells (H).
